# Primary Sjogren syndrome – A bibliometric analysis via CiteSpace

**DOI:** 10.1097/MD.0000000000038162

**Published:** 2024-06-14

**Authors:** Mingrui Yang, Shangzhi Wang, Jin Zhang, Bin Yan

**Affiliations:** a School of pharmacy, Shandong University of Traditional Chinese Medicine, Ji’nan, China; b School of Traditional of Chinese Medicine, Shandong University of Traditional Chinese Medicine, Ji’nan, China.

**Keywords:** bibliometric, CiteSpace, knowledge-map, primary Sjogren syndrome, Web of Science

## Abstract

This study employs CiteSpace software to analyze the research status, hotspots, and trends of primary Sjogren syndrome (pSS). Relevant publications from 1999 to 2023 were searched in the Web of Science Core Collection (WoSCC) set, followed by generating a network map using CiteSpace software to identify top authors, institutions, countries, keywords, journals, references, and research trends. A total of 3564 valid articles were included in this study. The People Republic of China had the highest number of articles (n = 524), while the University of Bergen emerged as the institution with the highest publication count (n = 94). Mariette X was identified as the author with the most publications (n = 67), whereas Vitali C received recognition as the most cited author (n = 1706). Annals of Rheumatic Diseases stood out as the journal with the highest citation count (n = 2530). Notably, an article published in the Annals of Rheumatic Diseases in 2017 garnered significant attention by being cited a remarkable 304 times. The bibliometric analysis reveals that key areas of research in pSS encompass investigating pathogenesis; advancing and applying targeted biological agents; and establishing treatment and diagnostic standards.

## 1. Introduction

The chronic autoimmune disease of unknown etiology, Sjogren syndrome (SS), was initially identified and named by Swedish ophthalmologist Hendrik Sjogren in 1933. It exhibits diverse clinical manifestations primarily affecting the lacrimal and salivary glands.^[1–3]^ Individuals who fulfill the diagnostic criteria for SS without concurrent autoimmune comorbidities are categorized as pSS patients, whereas those presenting with additional autoimmune comorbidities such as rheumatoid arthritis or systemic lupus erythematosus are classified as secondary Sjogren syndrome patients.^[4]^ In this review, we will focus on pSS.

In recent decades, researchers have conducted extensive and in-depth investigations on primary Sjogren syndrome (pSS), leading to significant advancements. However, a comprehensive systematic analysis of these research findings is yet to be undertaken.

The Java-based software tool, CiteSpace, utilizes metrology, co-occurrence analysis, and cluster analysis methods to visually map scientific knowledge. It effectively integrates mathematical, visual, and philosophical thinking to synthesize advancements in a research field and uncover focal areas of investigation within that domain. In comparison to other bibliometric software tools, CiteSpace offers an advantage in identifying emerging trends and elucidating their temporal evolution through timelines.

This study aims to provide valuable insights into pSS research by conducting a comprehensive analysis of the literature published in the past 2 decades. Utilizing CiteSpace, we performed a visual examination of pSS-related publications, elucidating its current research status and identifying emerging hot topics. Our findings aim to guide future developments in pSS research and offer direction for clinical investigations.

## 2. Method

### 2.1. Search strategy

**Figure 1.**
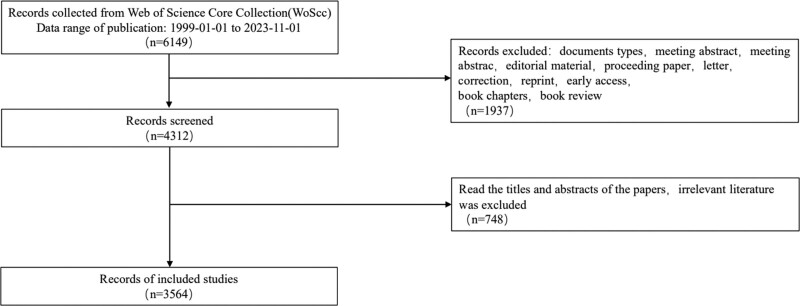
Flowchart of the screening process.

We retrieved articles in the Web of Science Core Collection (WoSCC) from 1999 to 2023, using pSS as terms. Inclusion and exclusion criteria peer-reviewed published original articles or review articles about the pSS were included. Exclusion criteria were conference abstracts or corrigendum documents; unpublished articles; repeated publications; unrelated articles (Fig. 1).

### 2.2. Bibliometrics and visualization analysis

We exported the retrieved article in plain text format with full records and citations as “download_XXX.txt” and imported it into CiteSpace 6.2.R5 for further analysis.

### 2.3. Literature research and analysis method

Employing innovative 6.2.R5, we conducted a comprehensive analysis of the existing literature on pSS, encompassing authorship, geographical distribution, institutional affiliations, and subject matter. These facets were compiled into a visual format to facilitate intuitive presentation, and they consistently unveil novel patterns over time, thereby deepening comprehension of significant areas of interest in the field. By adopting this approach, our aim is to foster profound understanding while providing valuable insights for future research endeavors.

## 3. Results

### 3.1. Analysis of publication year and journal

**Figure 2.**
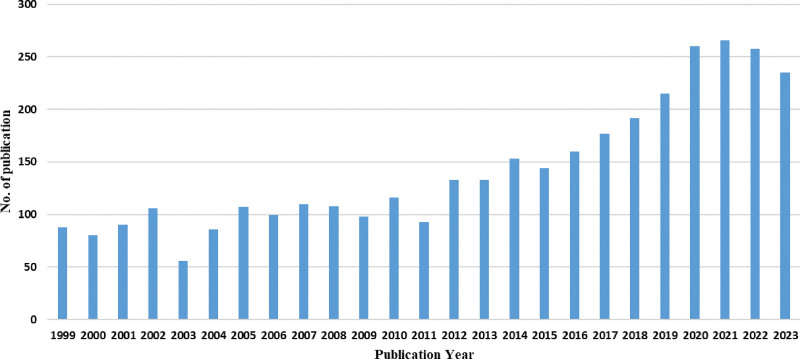
Trend chart of annual publications.

Since 1999, a total of 3564 papers on pSS have been included in the WoSCC, as depicted in Figure 2. The annual average number of publications reported from 1999 to 2023 was 148, with the highest count of 266 papers observed in 2021. This surge can be attributed to the global attention intensified by the COVID-19 pandemic toward pSS as an underlying disease, highlighting its emergence as a prominent research topic worldwide. Over the past 2 decades, pSS has emerged as a disease of significant incidence within the field of immunology, exhibiting a consistent growth trajectory in academic literature. This surge in published research signifies an augmented focus by scholars on exploring pSS with greater breadth and depth, paralleling advancements in modern medical expertise.

The findings of pSS research have been disseminated across a wide range of scholarly journals, totaling 743 in number. Table 1 provides an overview of the top 10 journals that have contributed significantly to the field of pSS research, offering valuable insights for researchers who are new to this domain. Notably, Clinical and Experimental Rheumatology emerges as the leading journal with a substantial publication count of 228 articles, focusing on elucidating the etiology, developmental mechanisms, epidemiology, and treatment modalities pertaining to immune diseases. Following closely is Clinical Rheumatology securing second place with an impressive publication count of 686 articles dedicated to clinical diagnosis and therapeutic interventions for Rheumatic Diseases.

**Table 1:** Top 10 journals by citation and publication.

Rank	Journal	Publications	IF (2023)	Rank	Journal	Citations	IF (2023)
1	Clinical and Experimental Rheumatology	228	3.7	1	Annals of the Rheumatic Diseases	2530	27.4
2	Clinical Rheumatology	138	3.4	2	Journal Of Rheumatology	1695	3.9
3	Rheumatology International	118	4	3	Arthritis and Rheumatism	1529	5
4	Journal of Rheumatology	116	3.9	4	Clinical and Experimental Rheumatology	1422	3.7
5	Frontiers in Immunology	105	7.3	5	Journal of Autoimmunity	1013	12.8
6	Annals of the Rheumatic Diseases	100	27.4	6	Journal of Immunology	961	4.4
7	Journal of Autoimmunity	70	12.8	7	Arthritis Research and Therapy	936	4.9
8	Arthritis and Rheumatism	62	5	8	Scandinavian Journal of Rheumatology	870	2.1
9	Autoimmunity Reviews	60	13.6	9	Autoimmunity Reviews	831	13.6
10	Arthritis Research and Therapy	54	4.9	10	Lancet	785	168.9

### 3.2. Analysis of authors

**Figure 3.**
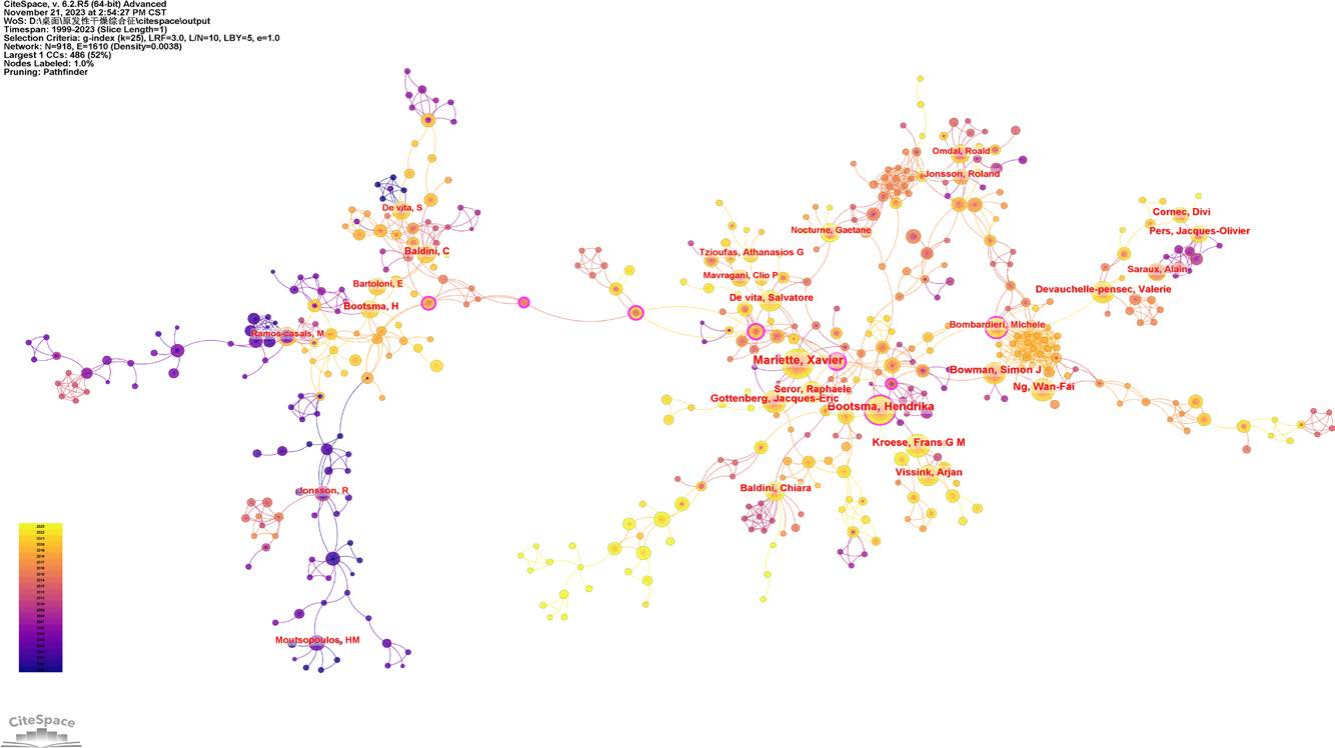
The network of co-authorship.

The co-authorship network shows prolific authors and the collaboration among them (Fig. 3). The size of the concentric circles represents the number of publications, and the more articles an institution publishes, the larger its concentric circles are. The connection between the 2 authors means that they publish together. The rough milling of the lines indicates the strength of their collaboration. Table 2 demonstrates that the most representative author in the field of pSS is Mariette X with a total of 67 published studies, followed by Bootsma H (61 articles) and Kroese FGM (48 articles). This analysis can provide highly personalized scientific research information for other researchers.

**Table 2:** Top 10 authors and co-cited authors.

Rank	Authors	Publications	Rank	Co-cited authors	Co-citations
1	Mariette X	79	1	VIitali C	1706
2	Bootsma H	66	2	Fox RI	760
3	Ng WF	46	3	Ramos M	709
4	Gottenberg JE	44	4	Seror R	602
5	Kroese FGM	43	5	Shiboski CH	491
6	Bowman SJ	41	6	Mariette X	471
7	Devauchell V	38	7	Gottenberg JE	412
8	Seror R	36	8	Theander E	409
9	De vita S	35	9	Brito ZP	378
10	Baldini C	35	10	Skopouli FN	316

### 3.3. Analysis of institution

**Figure 4.**
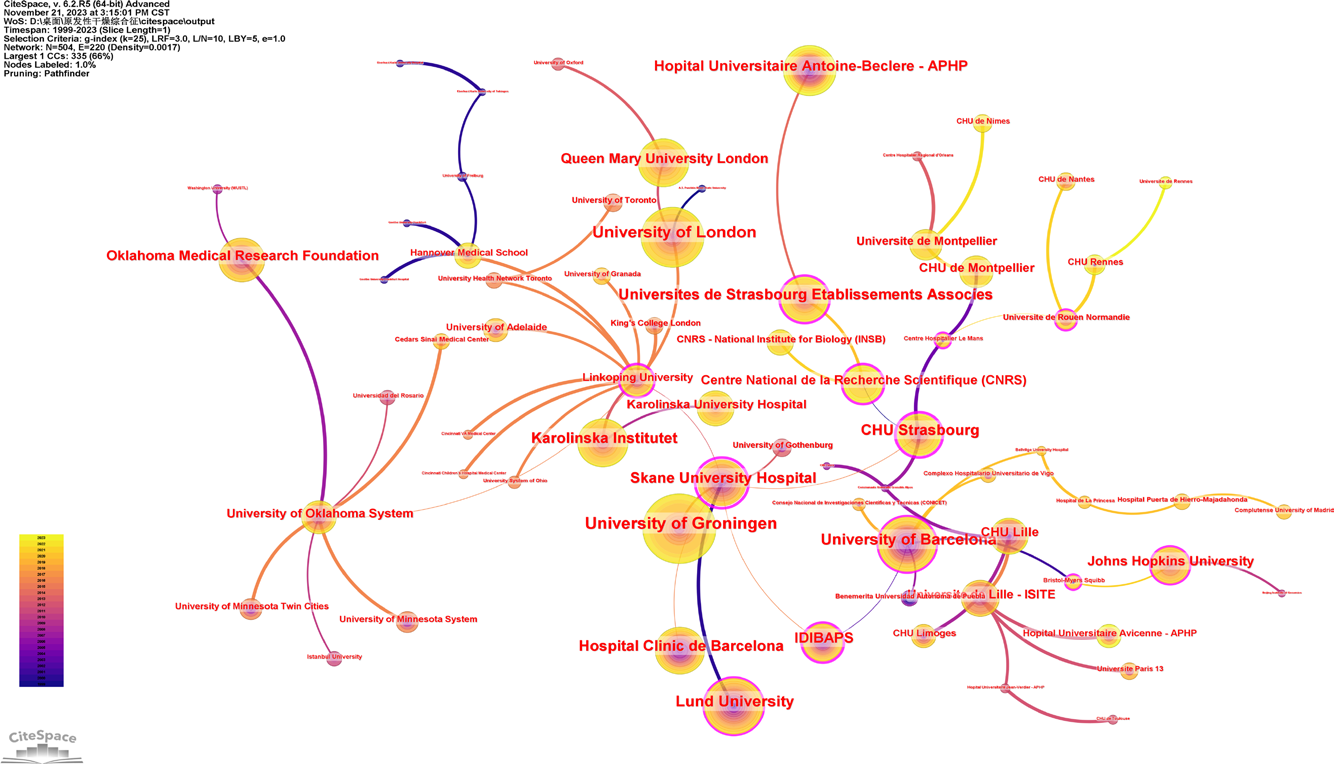
Co-occurrence map of institutions.

A total of 3163 institutions were involved in publishing pSS-related papers (Fig. 4). The top 5 institutions were the University of Bergen (94 articles), University of Groningen (94 articles), University of Athens (67 articles), University of Pisa (64 articles), and Johns Hopkins Hospital (52 articles).

### 3.4. Analysis of country

**Figure 5.**
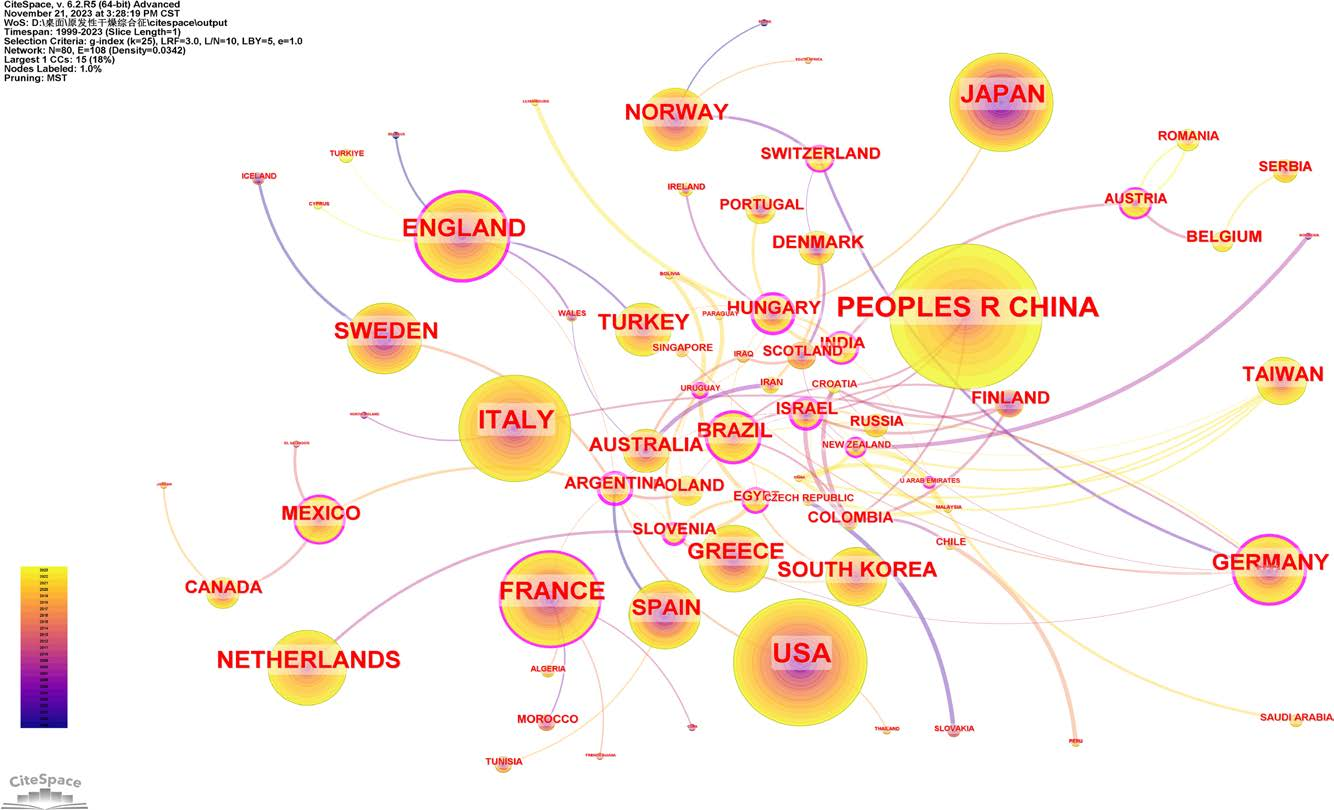
Co-occurrence map of country.

A total of 109 countries/regions have published papers on pSS. People Republic of China was the leading country (524 articles), followed by the United States (450 articles), Italy (316 articles), Japan (273 articles), and France (265 articles). Among them, the betweenness centrality of the United States is the highest, which is 0.25, indicating that the United States has the greatest contribution in this field (Fig. 5).

### 3.5. Analysis of references

**Figure 6.**
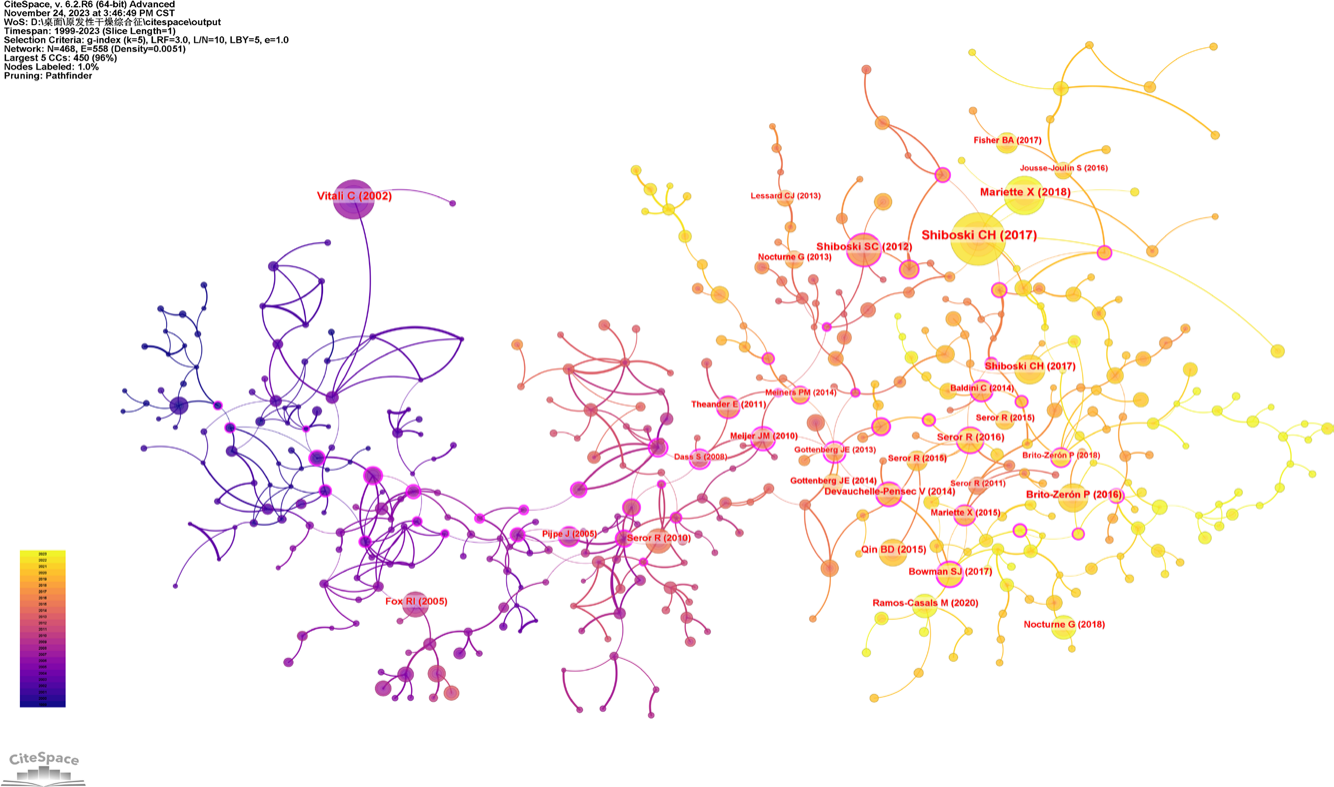
The network of co-cited references.

Figure 6 and Table 3 show the top co-cited references with high frequency. The first co-cited reference was published by Shiboski et al,^[5]^ which developed and validated an international set of classification criteria for pSS using guidelines from the American College of Rheumatology and the European League Against Rheumatism (EULAR).The reference published by Mariette et al discussed the diagnosis and treatment of pSS.^[6]^ Vitali et al^[7]^ validate and improve the European standards for pSS as revised by the American–European Consensus Group.

**Table 3:** The top 10 cited references.

Rank	First author	Country	Frequency	Cited references	Journal	Yr	IF
1	Shiboski CH^[5]^	USA	304	2016 American College of Rheumatology/European League Against Rheumatism classification criteria for primary Sjogren syndrome: A consensus and data-driven methodology involving 3 international patient cohorts	Annals of the Rheumatic Diseases	2017	27.4
2	Mariette X^[6]^	France	178	Primary Sjogren syndrome	New England Journal of Medicine	2018	158.5
3	Vitali C^[7]^	Italy	176	Classification criteria for Sjogren syndrome: a revised version of the European criteria proposed by the American–European Consensus Group	Annals of the Rheumatic Diseases	2002	27.4
4	Shiboski SC	USA	123	American College of Rheumatology classification criteria for Sjogren syndrome: A data-driven, expert consensus approach in the Sjogren International Collaborative Clinical Alliance Cohort	Arthritis Care and Research	2012	4.7
5	Shiboski CH^[5]^	USA	108	2016 American College of Rheumatology/European League Against Rheumatism Classification Criteria for Primary Sjogren syndrome: A Consensus and Data-Driven Methodology Involving 3 International Patient Cohorts	Arthritis and Rheumatology	2017	13.3
6	Brito-Zerón P	Spain	95	Sjogren syndrome	Nature Reviews Disease Primers	2016	81.5
7	Qin BD	Peoples R China	83	Epidemiology of primary Sjogren syndrome: a systematic review and meta-analysis	Annals of the Rheumatic Diseases	2015	27.4
8	Seror R	France	76	EULAR Sjogren syndrome disease activity index: development of a consensus systemic disease activity index for primary Sjogren syndrome	Annals of the Rheumatic Diseases	2010	27.4
9	Fox RI	USA	76	Sjogren syndrome	Lancet	2005	168.9
10	Bowman SJ	France	75	Defining disease activity states and clinically meaningful improvement in primary Sjogren syndrome with EULAR primary Sjogren syndrome disease activity (ESSDAI) and patient-reported indexes (ESSPRI)	Annals of the Rheumatic Diseases	2016	27.4

**Figure 7.**
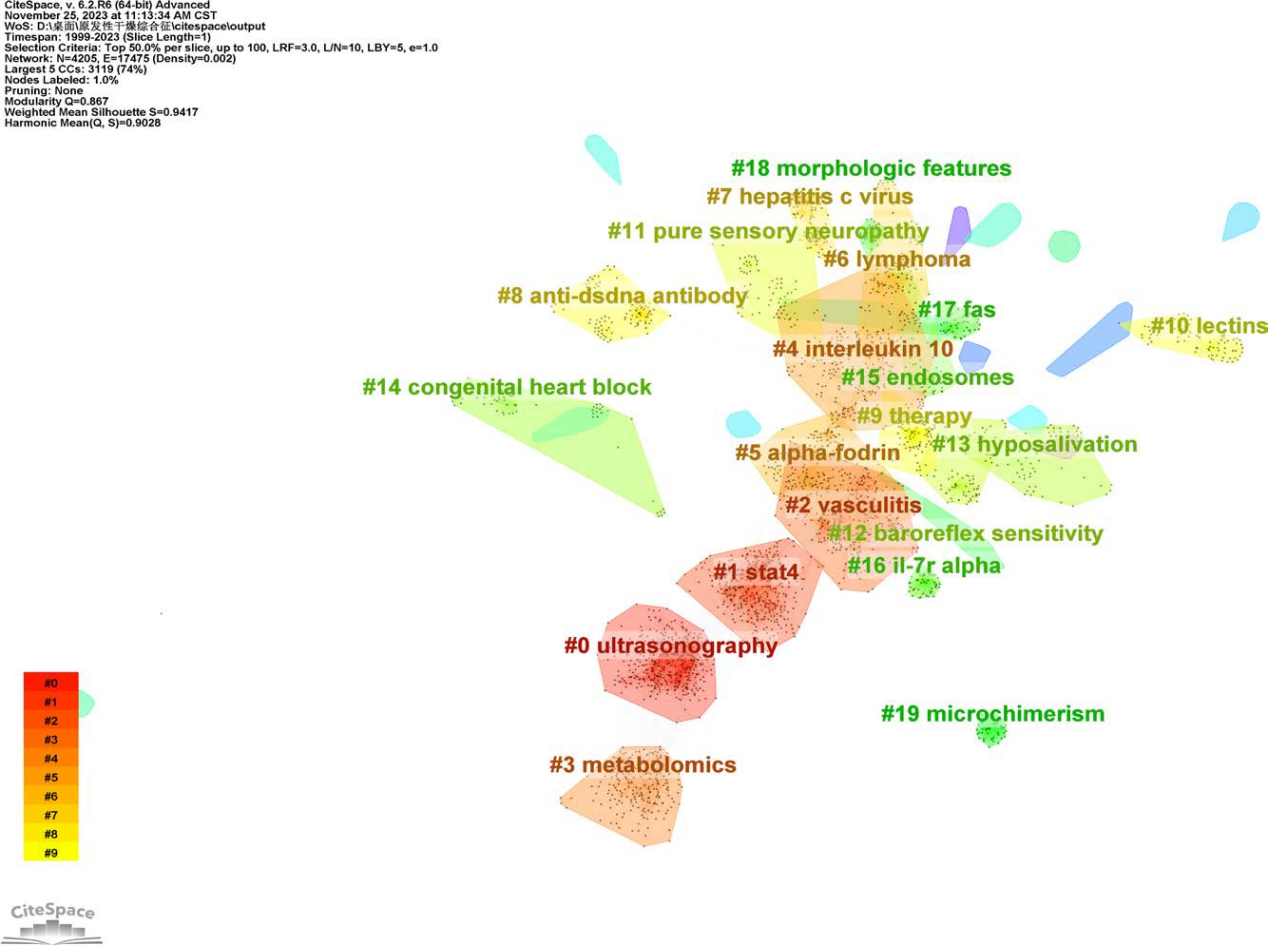
The network of co-cited references clusters.

**Figure 8.**
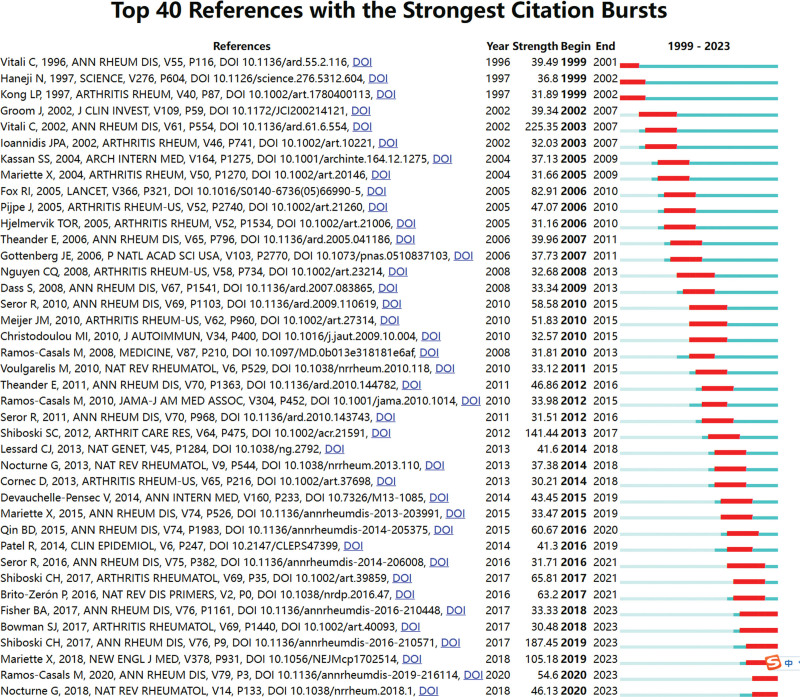
Top 25 references with the strongest citation bursts.

Figure 7 shows the largest 20 clusters of co-cited references. Figure 8 displays the top 25 references with the strongest citation bursts, which indicate the emerging trends or increasing interests in the field. Generally, the most co-cited references usually got the strongest citation bursts.^[8]^

### 3.6. Analysis of keywords

**Figure 9.**
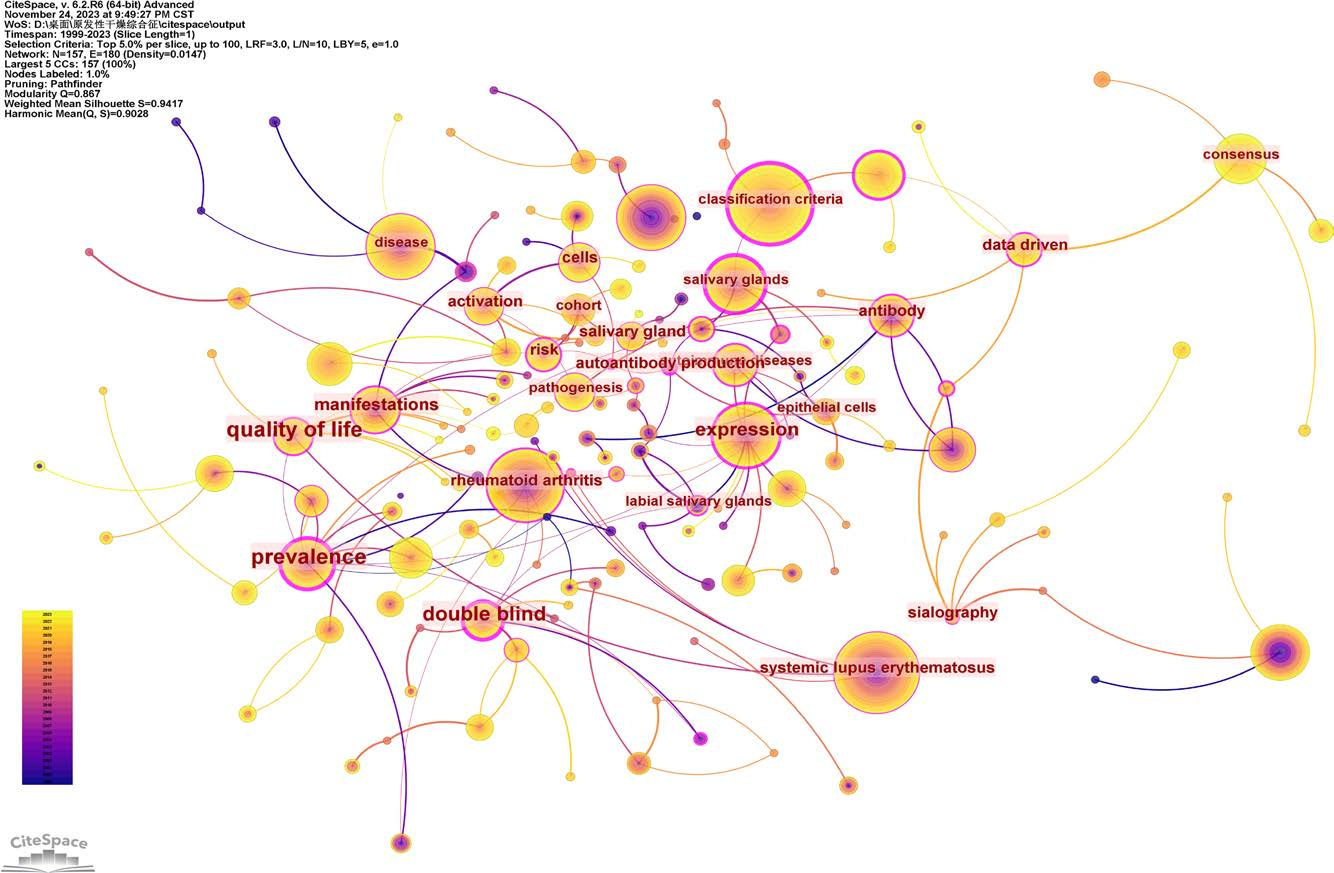
Analysis of co-occurring keywords.

**Figure 10.**
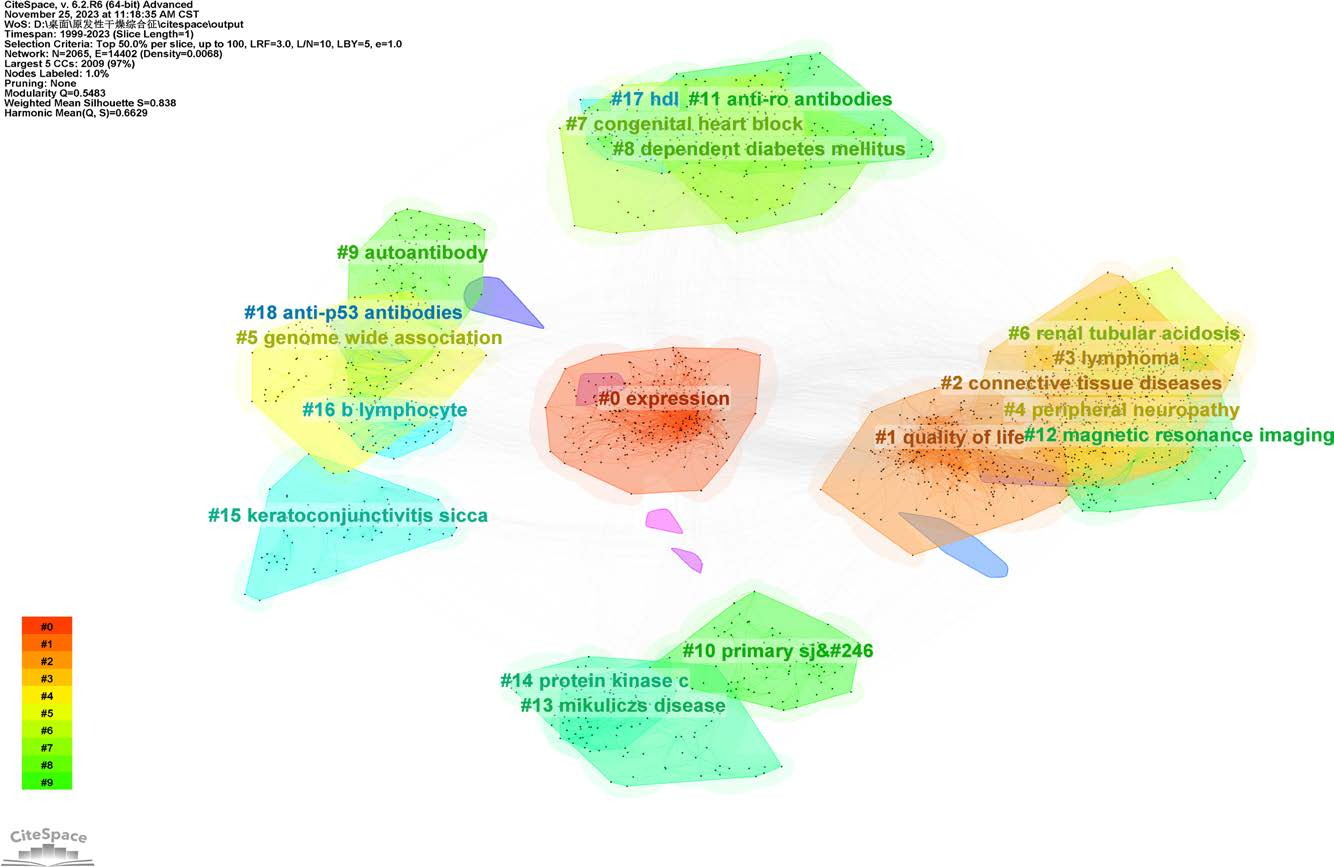
The network of co-occurring keyword clusters.

**Figure 11.**
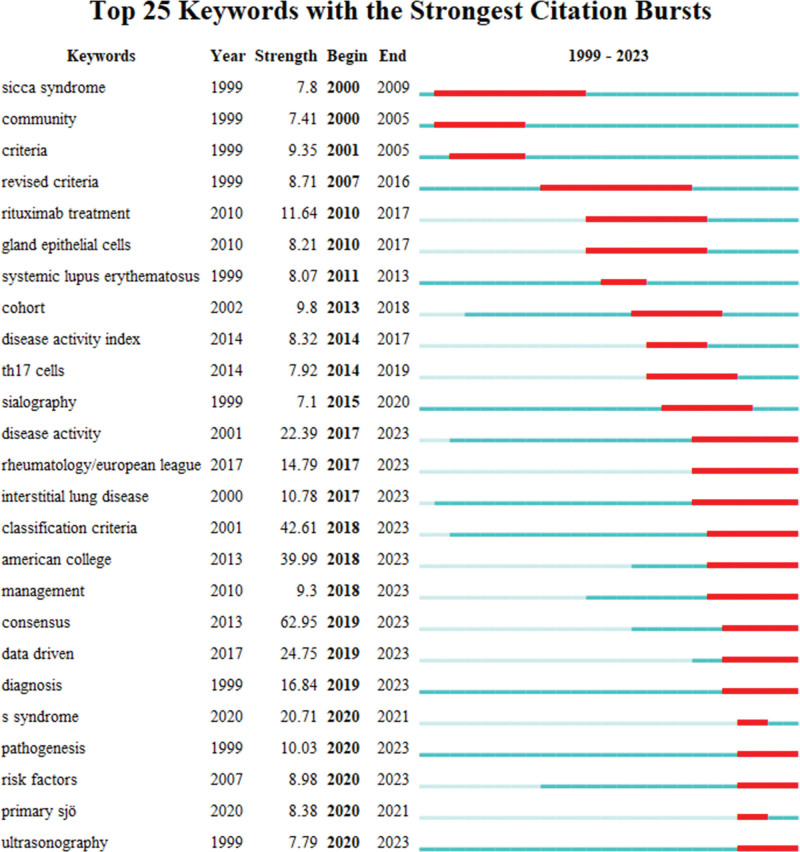
The top 25 keywords with citation bursts.

Keywords are high-level summaries. High-frequency and high-centrality keywords often reflect the research hotspot in this field (Fig. 9). Nodes represent keywords, and the size of each node corresponds to the co-occurring frequency of the keywords. The color of the lines that appear together between keywords indicates chronological order. Table 4 shows the top 10 high-frequency keywords and their centrality. Figure 10 and Table 5 show the largest 20 clusters of keywords. Figure 11 shows the top 25 keywords with citation bursts. The blue line indicates the time interval, while the red line indicates the time period when a keyword had a burst.^[9]^ Keywords “rituximab treatment” with the strongest citation bursts appeared in 2010, these results indicated the importance of rituximab in the treatment of pSS. The most recent keywords with citation bursts that occurred in 2019 were “consensus,” “data driven,” and “diagnosis.” In addition, “ultrasonography” and “risk factors” also continued to 2023.

**Table 4:** The top 10 keywords of frequency and centrality.

Rank	Frequency	Keywords	Centrality	Keywords
1	555	Systemic lupus erythematosus	0.06	Autoimmune diseases
2	492	Classification criteria	0.05	Malignant lymphoma
3	465	Rheumatoid arthritis	0.05	Autoimmunity
4	412	Disease	0.05	Patient
5	386	Classification	0.05	Diseases
6	345	Expression	0.04	Autoantibody
7	290	Salivary glands	0.04	Diagnosis
8	255	Criteria	0.04	B-cells
9	234	Prevalence	0.04	Lupus erythematosus
10	201	Manifestations	0.04	Autoimmune disease

**Table 5:** Keyword clustering information.

Cluster name	Number of nodes	Silhouette	Yr	Top terms (log-likelihood ratio, p-level)
#0 expression	306	0.642	2005	Expression (60.41, 1.0E−4); T-cells (47.31, 1.0E−4); activation (42.13, 1.0E−4); B-cells (41.88, 1.0E−4); epithelial cells (39.42, 1.0E−4)
#1 quality of life	284	0.673	2006	Quality of life (91.74, 1.0E−4); ultrasonography (74.72, 1.0E−4); fatigue (68.2, 1.0E−4); primary Sjogren syndrome (55.81, 1.0E−4); depression (51.31, 1.0E−4)
#2 connective tissue diseases	170	0.856	2002	Connective tissue diseases (64.76, 1.0E−4); systemic sclerosis (62.78, 1.0E−4); interstitial lung disease (45.54, 1.0E−4); connective tissue disease (38.76, 1.0E−4); pulmonary arterial hypertension (35.27, 1.0E−4)
#3 lymphoma	156	0.846	2002	Lymphoma (34.31, 1.0E−4); malignant lymphoma (26.22, 1.0E−4); cancer (24.64, 1.0E−4); mortality (22.14, 1.0E−4); autoimmune diseases (21.74, 1.0E−4)
#4 peripheral neuropathy	105	0.882	2001	Peripheral neuropathy (23.65, 1.0E−4); heart rate variability (21.45, 1.0E−4); neurological manifestations (21.45, 1.0E−4); autonomic dysfunction (17.01, 1.0E−4); sensory neuropathy (14.83, 0.001)
#5 genome wide association	95	0.886	2002	Genome wide association (38.03, 1.0E−4); susceptibility (32.35, 1.0E−4); variants (31.62, 1.0E−4); Sjogren syndrome (25.33, 1.0E−4); tnfaip3 (18.95, 1.0E−4)
#6 renal tubular acidosis	81	0.927	2002	Renal tubular acidosis (77.13, 1.0E−4); nephrocalcinosis (30.02, 1.0E−4); tubulointerstitial nephritis (30.02, 1.0E−4); renal involvement (24.01, 1.0E−4); case report (21.85, 1.0E−4)
#7 congenital heart block	79	0.897	2002	Congenital heart block (30.19, 1.0E−4); microarray (15.35, 1.0E−4); serum (11.58, 0.001); igg (11.58, 0.001); ro/ss-a (9.9, 0.005)
#8 dependent diabetes mellitus	79	0.903	2002	Dependent diabetes mellitus (16.54, 1.0E−4); la/ssb (12.76, 0.001); hla (11.63, 0.001); immune response (8.33, 0.005); apoptotic cells (8.26, 0.005)
#9 autoantibody	76	0.972	2002	Autoantibody (30.22, 1.0E−4); diseases (19.66, 1.0E−4); autoimmune pancreatitis (15.29, 1.0E−4); antimitochondrial antibody (12.27, 0.001); carbonic anhydrase ii (12.27, 0.001)
#10 primary sjö	74	0.961	2002	Primary sjö (47.64, 1.0E−4); s syndrome (38.09, 1.0E−4); hyposalivation (34.71, 1.0E−4); gren&apos (21.04, 1.0E−4); microorganism (16.99, 1.0E−4)
#11 anti-ro antibodies	72	0.945	2001	Anti-ro antibodies (10.75, 0.005); immunoreactive nerves (8.69, 0.005); primers (8.69, 0.005); scd 14 (8.69, 0.005); antidsdna antibody (8.69, 0.005)
#12 magnetic resonance imaging	70	0.928	2002	Magnetic resonance imaging (46.46, 1.0E−4); central nervous system (23.83, 1.0E−4); antigen epitopes (9.49, 0.005); human leukocyte antigen (9.49, 0.005); anca (9.49, 0.005)
#13 mikuliczs disease	65	0.914	2001	Mikuliczs disease (20.83, 1.0E−4); autoimmune epithelitis (13.06, 0.001); exocrine glands (13.06, 0.001); autoimmune pancreatitis (8.62, 0.005); differential diagnosis (8.62, 0.005)
#14 protein kinase c	62	0.939	2002	Protein kinase c (18.45, 1.0E−4); lymphocytic infiltration (18.45, 1.0E−4); nod mice (10.89, 0.001); endosomes (9.21, 0.005); human salivary acinar cells (9.21, 0.005)
#15 keratoconjunctivitis sicca	58	0.942	2001	Keratoconjunctivitis sicca (19.05, 1.0E−4); surface (17.74, 1.0E−4); psychosis (13.94, 0.001); fusion protein (13.94, 0.001); different age groups (8.86, 0.005)
#16 b lymphocyte	49	0.964	2002	B lymphocyte (13.06, 0.001); igv gene usage (10.66, 0.005); blys (10.66, 0.005); lymphocyte stimulator (10.66, 0.005); develop (10.66, 0.005)
#17 hdl	32	0.98	2002	HDL (10.72, 0.005); cholesterol (10.72, 0.005); parotid gland swelling (10.72, 0.005); c-reactive protein (10.72, 0.005); glycoconjugates (10.72, 0.005)
#18 anti-p53 antibodies	14	0.995	2000	Anti-p53 antibodies (12.86, 0.001); sjogrens disease (12.86, 0.001); p21 (12.86, 0.001); p53 mutations (12.86, 0.001); p53 (12.86, 0.001)
#19 bickerstaffs brainstem encephalitis	12	1	2004	Bickerstaffs brainstem encephalitis (13.21, 0.001); cisplatin (13.21, 0.001); miller fisher syndrome (13.21, 0.001); dysimmune sensory neuropathy (13.21, 0.001); toxic neuropathy (13.21, 0.001)

## 4. Discussion

### 4.1. Summary of findings

This study provides a visual analysis of research on pSS from 1999 to 2023 by CiteSpace software. A total of 3564 related articles were retrieved eventually. The number of published articles on the topic increased rapidly after 2015, reaching more than 200 annually in 2019. Over the past 2 decades, more and more researchers have begun to study the field of pSS, forming a prolific group of authors represented by Mariette, Bootsma, and Ng. USA was the leading country in this field and USA institutions have published many relevant studies. The most cited authors and references generally focus on the classification criteria for pSS. Top 5 keywords with both high frequency were systemic lupus erythematosus, classification criteria, rheumatoid arthritis, disease and classification, while the investigation of pathogenesis, the advancement and application of targeted biological agents, and the establishment of treatment and diagnostic standards constitute the focal points of research in pSS. These results can help guide future research. Researchers new to the field can easily obtain useful and relevant information from our bibliometric analysis.

### 4.2. Research hotspots on pSS

#### 4.2.1. Pathogenesis

pSS is a systemic autoimmune disease characterized by lymphocyte infiltration of secretory glands, especially lacrimal and salivary glands. The pathogenesis of pSS is not clear, it involves the intricate interplay of multiple factors, which is widely acknowledged to be intricately linked to genetic susceptibility, environmental influences, and immunological dysregulation. Increased B-cell activation, subsequent formation of immune complexes and autoantibody production are considered to play an important role in the course of pSS.^[10, 11]^ Studies have indicated that the pathogenesis of pSS arises from the intricate interplay between the immune system and epithelial cells, which serve as both targets and active participants in autoimmune responses.^[12]^ Under the influence of genetic predisposition and environmental factors, critical epithelial cell pathways such as Janus-activated kinase/signal transducers and activators of transcription and epithelial growth factor signaling are activated, resulting in excessive accumulation and activation of immune cells including dendritic cells, B-cells, and T-cells. Through the assistance of chemokine and adhesion factors, these immune cells migrate to the glands where they induce a variety of pro-inflammatory factors to activate adjacent epithelial cells. One specific type of excessively accumulated dendritic cell can produce a high concentration of interferon (IFN)-α, which stimulates epithelial cells, T-cells, and dendritic cells to generate B-cell activating factor. B-cell activating factor then promotes the maturation of abnormal B-cells within lymphatic germinal centers leading to autoantibody production and subsequent development of autoimmunity.^[13]^

#### 4.2.2. Diagnostic

PSS is a complex disease with diverse manifestations, progression patterns, and outcomes. However, there is no definitive clinical, laboratory, pathological, or radiological feature that can be considered as the ultimate diagnostic criterion or classification standard for this syndrome.^[14]^ Numerous classification criteria have been established since the 1970s. The diagnosis of pSS relies on characteristic clinical manifestations and symptoms (Table 6), in addition to specific examinations encompassing salivary gland histology and autoantibody testing. It is important to emphasize that the diagnosis of pSS necessitates a comprehensive approach, encompassing careful consideration of patient symptoms, thorough physical examination, meticulous laboratory investigation, and precise tissue biopsy.

Dryness represents the principal symptom of pSS, although certain patients may exhibit additional manifestations, including fatigue, arthralgia, and arthritis, or extra glandular manifestations such as lymphoma. pSS exhibits a lower prevalence in males and primarily affects elderly females.^[14, 25]^

Salivary glands represent the primary target organs of pSS, thereby effectively reflecting the potential autoimmune exocrine glandular lesions associated with this condition. Salivary gland ultrasonography plays a crucial role in the early diagnosis and assessment of pSS, providing essential information for understanding salivary gland lesions.^[26]^ In comparison with traditional sialography and scintigraphy, salivary gland ultrasonography represents a noninvasive modality devoid of ionizing radiation exposure, allowing for repetitive examinations while being well-suited for outpatient settings.^[27]^

The production of antinuclear antibodies occurs in approximately 70% to 90% of patients diagnosed with pSS. Notably, the presence of anti-Ro/SSA and anti-La/SSB antibodies demonstrates high specificity for diagnosing pSS. Additionally, serum testing can accurately determine the levels of anti-SS-A and anti-SS-B antibodies, which serve as specific markers for pSS.^[28]^ Antibodies against parotid secretory protein, carbonic anhydrase 6 (CA6), and salivary protein 1 (SP1) are considered potential biomarkers for early-stage pSS, while anti-rgi2, antienolase, and anti-cofilin-1 antibodies can serve as biomarkers for mucosal-associated lymphoid tissue lymphoma.^[29, 30]^

#### 4.2.3. Treatment

Currently, the management of pSS primarily focuses on symptomatic treatment, with no definitive solution identified thus far. The drugs utilized for the management of pSS can be categorized into the subsequent 3 groups:

##### 4.2.3.1. Relieving dry skin and mucous membranes

Such as artificial tears and gargle. Dry mouth and dry eyes are the most prevalent and distressing symptoms experienced by patients diagnosed with pSS. The administration of artificial tears and gargle solutions has been proven to provide prompt and effective relief for individuals suffering from these symptoms. Artificial tears not only alleviate ocular discomfort but also contribute to corneal hydration while mitigating inflammatory mediators. Furthermore, gargling not only aids in lubrication but also diminishes oral complications such as oral ulcers and dental caries.^[31–33]^

##### 4.2.3.2. Immunosuppressant

It belongs to a class of drugs that inhibit the immune response within the body by suppressing proliferation and function of immune-related cells (primarily T-cells and B-cells), thereby reducing overall immune activity. Iguratimod (IGU) is a small molecule compound that is widely used in China and Japan as a potential antirheumatic drug for the treatment of various Rheumatic Diseases.^[34]^ The inhibitory effect of IGU on B-cell function is mediated through the reduction in immunoglobulin production and the suppression of various inflammatory cytokines, such as interleukin (IL)-1, IL-6, IL-8, and tumor necrosis factor.^[35]^ Because of its multiple immunomodulatory effects, IGU has become a widely used drug for the treatment of Rheumatic Diseases, including pSS. Hydroxychloroquine (HCQ) is an immunomodulatory drug widely used in pSS, especially for patients with fatigue. Because of its multiple immunomodulatory effects, IGU has become a widely used drug for the treatment of Rheumatic Diseases, including pSS. The potential mechanism of HCQ in the treatment of pSS can be elucidated from 2 perspectives: at the pharmacological level, HCQ exhibits a propensity to accumulate within acidic compartments such as lysosomes and inflammatory tissues; and at the cellular level, HCQ exerts immunomodulatory effects by inhibiting autophagy processes, thereby preventing immune activation across various cell types, regulating CD154 expression on T-cells, and suppressing cytokine production.^[36–38]^

##### 4.2.3.3. Biological agents

Compared with traditional antirheumatic drugs, biological agents have the characteristics of quick onset, good patient tolerance and small adverse reactions. According to distinct targets, biological agents can be categorized into drugs targeting B-cells, stem cells, T-cells and cytokines.

B-cells play a pivotal role in the pathogenesis and progression of pSS due to their hyperactivity, which triggers germinal center formation and autoantibody production. Furthermore, the presence of autoantibodies, hyperglobulinemia, and increased risk of non-Hodgkin lymphoma underscore the central involvement of B-cells in pSS pathogenesis. Consequently, targeting B-cells has emerged as a principal approach for enhancing treatment efficacy in pSS.^[39, 40]^ Rituximab (RTX) is a chimeric antibody that specifically binds to the CD20 antigen expressed on the majority of B-cell progenitors, thereby facilitating their activation, proliferation, and differentiation. Additionally, it effectively reduces the population of circulating B-cells through complement-dependent cytotoxicity and antibody-dependent cytotoxicity.^[41]^ Chen et al demonstrated that RTX effectively ameliorates symptoms in patients with pSS by depleting peripheral B-cells. However, the duration of therapeutic response is limited and relapse often occurs upon drug discontinuation. This relapse may be attributed to the reconstitution of CD20^+^ B-cell population to pretreatment levels. Therefore, further investigations are warranted to identify suitable candidates for RTX treatment and optimize therapeutic strategies for enhanced efficacy. Moreover, RTX administration carries potential side effects such as infection and immunosuppression; however, these risks can be mitigated by adjunctive intravenous immunoglobulin (IVIg) therapy. Overall, monotherapy with rituximab fails to fully restore immune function in patients necessitating additional research efforts to enhance its therapeutic effectiveness.^[42]^

Mesenchymal stem cells (MSCs) are a class of cells with self-renewal and differentiation potential, which are derived from bone marrow, umbilical cord, gingival and adipose tissue.^[43]^ MSCs exert immunomodulatory effects, influencing both adaptive and innate immune responses. They impede T-cell proliferation and activation through direct cellular contact and the release of soluble factors, regulate T-cell differentiation, and impact B-cell activation as well as antibody production. Furthermore, MSCs also modulate the activation and function of dendritic cells, macrophages, and natural killer cells. These immunomodulatory properties have demonstrated potential therapeutic benefits in animal models and patients with pSS. However, challenges persist in utilizing MSCs for pSS treatment due to functional deficiencies and inconsistent therapeutic outcomes. Therefore, further research efforts coupled with enhanced techniques may enhance the efficacy of MSC-based therapies for pSS.^[44–46]^

**Table 6:** The primary symptoms of primary Sjogren syndrome.

Position	Symptom
Oral cavity	Dryness of the mouth and throat, thirst, mouth pain, difficulty swallowing; mouth ulcers^[^^15^^]^
Ocular	Dry eyes, pain, fatigue, blurred vision, light sensitivity^[^^16, 17^^]^
Other dry symptoms	Dry nose, stuffy nose, hoarseness, dry skin, dry vagina^[^^18, 19^^]^
Joint	Joint pain, joint swelling, joint stiffness, joint limitation^[^^20^^]^
Systemic manifestations	Chronic fatigue, general weakness, muscle aches, weight loss^[5]^
Cutaneous	Rash, dry skin, purpura^[^^21^^]^
Digestive system	Loss of appetite, indigestion, diarrhea or constipation^[^^22^^]^
Urinary system	Frequent urination, urgent urination, painful urination^[^^23, 24^^]^

## 5. Novelty

Compared to previous bibliometric analyses of pSS, our study includes relevant literature from 1999 to 2023 in the WoSCC and carefully screens it, eliminating a significant amount of irrelevant literature. As a result, our analysis is more detailed and comprehensive than previous studies. Additionally, we conduct specific analyses on author cooperation networks and institution cooperation networks while also performing cluster analyses on keywords. These efforts allow new researchers in this field to become more familiar with research hotspots and frontier directions.

## 6. Limitations

There are still some limitations to our study: Recently published articles may exhibit limited citation counts due to the constrained timeframe for citations, thereby potentially introducing research bias into the study. This study exclusively considered articles and reviews published in the English language, potentially disregarding relevant literature written in other languages. Our literature search was limited to WoSCC, which may not encompass all studies in the field of pSS, introducing potential bias into our findings.

## 7. Outlooking

PSS follows a protracted course, and although its pathogenesis remains incompletely elucidated, it is closely associated with the dysfunction of T lymphocytes, B lymphocytes, and NK cells. Furthermore, viral infections and genetic factors are also implicated in the disease etiology. Currently, the precise pathogenic mechanisms remain undisclosed, posing challenges for developing a unified treatment plan. Therefore, optimizing clinical management based on further exploration becomes an imperative topic of interest. Presently, immunosuppressants, corticosteroids, and targeted drugs are employed to control disease progression and reduce activity levels. However, significant interindividual variations exist in drug application necessitating comprehensive investigations to address adverse reactions and other related concerns. With enhanced comprehension of relevant mechanisms alongside advancements in novel drug development and implementation strategies; we anticipate that future treatment approaches for patients with pSS will move toward precision-based interventions tailored to individual needs.

## Author contributions

**Conceptualization:** Mingrui Yang, Yan Bin.

**Data curation:** Mingrui Yang.

**Formal analysis:** Mingrui Yang.

**Methodology:** Mingrui Yang, Shangzhi Wang, Jin Zhang.

**Software:** Mingrui Yang.
